# Fifth District Dental Society

**Published:** 1885-05

**Authors:** 


### FIFTH DISTRICT DENTAL SOCIETY.

The seventeenth annual meeting of the Fifth District Dental Society was held at the city of Utica, on Tuesday and Wednesday, April 7 and 8, 1885, Dr. C. E. Cherry, of Syracuse, presiding.

The annual address was delivered by Dr. Cherry, and papers were read by Drs. G. L. Curtis, E. L. Swartwout, A. Retter, and J. C. House.

Three cases of illegal practicing were reported to the society, and the proper steps were taken to stop such violations of the law.

Three members were charged with advertising in a manner deemed in violation of the code of ethics and rules of the society, and the secretary was instructed to advise such members that, unless they discontinued such advertising, their names would be presented at the next semi-annual meeting for expulsion.

The society accepted an invitation to hold a union session with the Sixth District Society, at Binghamton, in October next.

The following were elected officers for the ensuing year:

*President*—C. C. Smith, Ilion.

*Vice-President*—G. L. Curtis, Syracuse.

*Recording Secretary*—C. J. Peters, Syracuse.

*Correspondent*—B. T. Mason, Phœnix.

*Treasurer*—J. C. House, Lowville.

*Librarian*—A. Retter, Utica.

*Censors*—S. B. Palmer, Syracuse, Charles Barnes, Syracuse, E. L. Swartwout, Utica.

